# Meetings of Societies

**Published:** 1914-03

**Authors:** 


### MEETINGS OF SOCIETIES.

### Bristol Medico-Chirurgical Society.

*December* 10*th*, 1913.

Dr. P. Watson-Williams, President, in the Chair.

The meeting was devoted to an **Exhibition of cases and specimens of Syphilis.**—Mr. Elwin Harris showed a child with chronic tertiary ulceration of the scalp.—Dr. J. O. Symes showed a patient with multiple swellings in and on the tibia and other bones, with a protein body in the urine. The patient was improving with antisyphilitic treatment; the Wassermann reaction was positive.—Dr. E. C. Williams stated that an infant, aged 5 months, with a gumma of the femur, which he was to have shown, had died forty-one days after an injection (intramuscular) of 0.05 gram of neo-salvarsan.—Dr. Carey Coombs, in showing a case of associated tabes dorsalis and aortic regurgitation, remarked that this was the not uncommon Babinski syndrome, and that syphilis was probably the cause of aortic disease in 75 per cent. of cases.—Dr. Bertram Rogers showed an infant, 2½ months old, in whom all symptoms had cleared up and the child put on weight after an intramuscular injection of 0.05 gram of neo-salvarsan.—Dr. Watson-Williams showed patients illustrating typical tertiary syphilitic lesions of the nose, pharynx and larynx.—Dr. Michell Clarke showed a case of associated congenital syphilis and Graves's disease, and said that he regarded the simultaneous occurrence as accidental.—Dr. J. R. Charles showed a case in which there were symptoms of tabes in association with plumbism; a positive Wassermann reaction was present, but might, it was thought, possibly be due to the lead.—Dr. J. R. Kay-Mouat, among other specimens, showed one of the site of injection of salvarsan nineteen days after injection. The tissues showed the presence of arsenic in the centre with a surrounding area of hyperæmia and leucocytic infiltration.

---

*January 14th*, 1914.

Dr. P. Watson-Williams, President, in the Chair.

Dr. Munro Smith read notes on **Two Cases of Abdominal Tumour in the Neighbourhood of the Liver, Plainly Palpable and Visible during Life, which disappeared at Death.** The first case was that of a lady of 61, who began to feel unwell on September 30th, 1910, with nausea and weakness. By October 16th she was jaundiced slightly. She gradually became weaker, and the question of the presence of malignant disease of the liver arose. By November 16th the liver edge was two inches below the thoracic margin, and a nodule was felt in it. Jaundice deepened. The diagnosis of liver enlargement, probably cancerous, was confirmed by three consultants. The patient died on January 11th, 1911. An autopsy showed the liver to be small and soft, and tucked up under the ribs. No abdominal tumour or swelling present. The heart muscle was soft and pliable. The second case was very similar. The patient was a man of 71. Malignant disease of the liver was diagnosed. No tumour was palpable after death, though distinct and nodular before death. No autopsy.—Dr. James Swain alluded to the simulation of tumours by reflex contraction of segments of the rectus muscle.

Dr. Symes showed a specimen of **A dilated stomach showing the pyloric opening in the middle of the lesser curvature. Ulceration at the pyloric orifice.**

Dr. Fortescue-Brickdale showed a **Case of combined sclerosis.** Woman aged 36. Symptoms began last June and have progressively increased. There are now spasticity, ankle and patellar clonus, and extensor plantar reflexes on both sides. Some weakness of right leg with anæsthesia, paræsthesia and analgesia. No muscular wasting. Arms and trunk normal.

Mr. HARTY showed a case of **Acute mastoiditis involving the outlying cells alone.** Symptoms: ear-ache for three weeks, no aural discharge, no signs of any perforation of membrana tympani. Subperiosteal abscess incised, antrum not opened. Recovery. (From the Ear, Nose and Throat Clinic of the Bristol Royal Infirmary, by kind permission of Dr. Watson-Williams.)

Dr. WATSON-WILLIAMS showed **Two patients on whom the intranasal frontal sinus operation has been performed after the method described by the exhibitor.** Entry to the fronto-nasal duct is gained by opening up and removal of the fronto-ethmoidal cells from a point corresponding to the anterior attachment of the middle turbinal, instead of by removal of the anterior end of the turbinal. A very free opening into the frontal sinus has been obtained in this way, thus ensuring efficient drainage without resorting to external operation.

---

*February 11th, 1914 (Afternoon Meeting).*

Dr. P. WATSON-WILLIAMS, President, in the Chair.

The meeting was devoted to **Demonstrations of Clinical Methods,** as follows:—

**Electric Cardiography,** by Professor STANLEY KENT and Dr. CAREY COOMBS.

**Gas, Oxygen, Ether Apparatus for Anoci-association,** by Mr. S. V. STOCK.

**The Estimation of Percentages of Ether Vapour in Air, and the Estimation of the Temperature of Anæsthetic Vapours and Saline Solutions, with an Electrical Thermometer,** by Mr. S. V. STOCK and Mr. J. D. FRY.

**A Modification of Lambotte's Apparatus for the Surgical Treatment of Fractures,** by Mr. C. F. WALTERS and Mr. H. W. GOODDEN.

**Anæsthetic Methods,** by Mr. A. L. FLEMMING.

**Oesophagoscopy and the Technique of Radium Therapy in Malignant Strictures; and Intruments and the Methods of Intranasal Operation for Frontal Sinus Suppuration,** by Dr. P. WATSON-WILLIAMS.

**Insects as Carriers of Disease, and Methods of Preserving and Mounting Specimens,** by Professor WALKER HALL and Dr. J. R. KAY-MOUAT.

**The use of Maddox's Phorometer and Schiotz's Tonometer,** by Mr. C. H. WALKER.

**Methods of Estimating Blood Pressure,** by Dr. J. M. FORTESCUE-BRICKDALE.

J. LACY FIRTH.
A. J. WRIGHT, *Hon. Sec.*

---

